# Computed Tomography Angiography in the Catheterization Laboratory: A Guide Towards Optimizing Coronary Interventions

**DOI:** 10.3390/jcdd12010028

**Published:** 2025-01-16

**Authors:** Eirini Beneki, Kyriakos Dimitriadis, Nikolaos Pyrpyris, Alexios Antonopoulos, Konstantinos Aznaouridis, Panagiotis Antiochos, Christos Fragoulis, Henri Lu, David Meier, Konstantinos Tsioufis, Stephane Fournier, Constantina Aggeli, Georgios Tzimas

**Affiliations:** 1 First Department of Cardiology, School of Medicine, National and Kapodistrian University of Athens, Hippokration General Hospital, 115 27 Athens, Greece; e.beneki@hotmail.com (E.B.); npyrpyris@gmail.com (N.P.); antonopoulosal@yahoo.gr (A.A.); conazna@yahoo.com (K.A.); christosfragoulis@yahoo.com (C.F.); ktsioufis@gmail.com (K.T.); dina.aggeli@gmail.com (C.A.); 2 Department of Cardiology, Lausanne University Hospital and University of Lausanne, 1011 Lausanne, Switzerland; panagiotis.antiochos@chuv.ch (P.A.); henri.lu@chuv.ch (H.L.); david.meier@chuv.ch (D.M.); stephane.fournier@chuv.ch (S.F.); georgios.tzimas@chuv.ch (G.T.)

**Keywords:** coronary angiography, computed tomography, atherosclerosis, coronary plaque, percutaneous coronary intervention, pre-procedural planning

## Abstract

Cardiac computed tomography (CT) has become an essential tool in the pre-procedural planning and optimization of coronary interventions. Its non-invasive nature allows for the detailed visualization of coronary anatomy, including plaque burden, vessel morphology, and the presence of stenosis, aiding in precise decision making for revascularization strategies. Clinicians can assess not only the extent of coronary artery disease but also the functional significance of lesions using techniques like fractional flow reserve (FFR-CT). By providing comprehensive insights into coronary structure and hemodynamics, cardiac CT helps guide personalized treatment plans, ensuring the more accurate selection of patients for percutaneous coronary interventions or coronary artery bypass grafting and potentially improving patient outcomes.

## 1. Introduction

Coronary computed tomography angiography (CCTA) represents a well-established and reliable modality for the clinical assessment and diagnosis of coronary artery disease (CAD), offering the advantages of non-invasiveness, accessibility, and highly sensitivity [1]. Current guidelines underscore the role of CCTA as a first-line test for evaluating patients with symptoms suggestive of obstructive CAD [2]. While CCTA is primarily a diagnostic tool, its utility extends beyond the initial diagnosis. It provides additional clinical insights that can inform decisions regarding referrals for invasive coronary angiography (ICA) and the planning of coronary interventions. Unlike structural heart interventions, where detailed pre-procedural imaging is routine, patients undergoing catheterization for coronary interventions often lack comprehensive, patient-specific data on coronary anatomy and lesion characteristics (length, diameter, degree of calcification) that could optimize procedural strategies.

As a result, the number of patients referred to the catheterization laboratory following a non-invasive evaluation with CCTA is anticipated to rise in the coming decade. While this diagnostic strategy has not yet been fully integrated into routine clinical practice, utilizing CCTA to assess patients being considered for invasive procedures could enhance the efficiency of catheterization laboratory operations and improve procedural planning. For the interventional cardiologist, CCTA has the potential to guide many aspects of intervention, which will undoubtedly aid patient selection, streamline cardiac catheterization laboratory workflow, and improve diagnostic performance and procedural safety. This review provides practical recommendations on the implementation of CCTA in the catheterization laboratory to guide coronary interventions and highlights the pivotal role of CCTA in guiding cardiologists in routine clinical practice.

## 2. Anatomical Evaluation of Coronary Arteries

Once significant CAD is identified, CCTA offers interventional cardiologists valuable additional insights for planning percutaneous coronary intervention (PCI). Advances in CCTA technology, including improvements in multi-slice computed tomography scanners, enable a more comprehensive evaluation of coronary anatomy, plaque composition, and calcium burden.

CCTA plays a critical role in managing complex lesions, such as unprotected left main coronary artery stenosis. Identifying left main disease prior to an invasive procedure can aid in selecting the appropriate revascularization strategy, facilitate patient consent, and provide insights into the potential requirement for hemodynamic support devices. Based on the degree and extent of left main stenosis, the clinician may opt for a specific PCI strategy, coronary artery bypass grafting (CABG) along with intensive medical therapy.

Understanding the location and orientation of the coronary ostia can aid in invasive catheterization. Noting that the RCA ostium is anterior may indicate the need for a different catheter type, such as an Amplatzer catheter, which helps engage the coronary artery and provides better support during PCI. Additionally, a left main ostium with a very posterior takeoff may require extra counter-clockwise torque, especially when approaching from the right radial artery, to achieve effective engagement. CCTA can provide the optimal fluoroscopic angulations of coronary artery ostia by providing a detailed, three-dimensional reconstruction of the heart. The ability to generate complete post-processing reconstructions also enhances the effectiveness of coronary interventions [3].

A significant advantage of CCTA is its ability to visualize both the vessel lumen and wall structure, similar to intravascular ultrasound (IVUS) [4]. While optical coherence tomography (OCT) and IVUS offer detailed insights into lesion characteristics and post-PCI stent outcomes, their invasive nature, high costs, and time-intensive processes limit their widespread use. In contrast, CCTA provides a non-invasive alternative, delivering comprehensive anatomic and quantitative coronary analysis, including detailed evaluations of plaque characteristics, luminal dimensions, and lesion length. For example, in heavily calcified lesions, plaque modification with rotational atherectomy or rotablation can facilitate the delivery of stents, improving procedural success. This positions CCTA as a viable substitute for these invasive imaging techniques [5].

CT-derived quantitative coronary analysis has demonstrated greater accuracy in determining true luminal dimensions compared to conventional angiography [6]. Incorporating CT into the planning phase of coronary interventions helps optimize angiographic projections in the catheterization laboratory, reducing foreshortening and minimizing overlap of the target segment. Bifurcation lesions assessed during ICA may hinder accurate assessment of the coronary anatomy and lead to underestimation of the dimensional nature of the stenotic lesion, resulting in vessel overlap, foreshortening, and beam attenuation. This is especially crucial for assessing the side-branch ostium in bifurcation lesions. Using CCTA and three-dimensional (3D) reconstruction of the coronary tree enables operators to visualize the entire coronary anatomy from any angle, eliminating overlap and foreshortening. It also provides detailed insights into vessel wall architecture and bifurcation angles before the patient undergoes PCI. This comprehensive evaluation can enhance the assessment of bifurcation lesions, allowing the operator to determine the extent of involvement in both the main and side branches and plan the appropriate strategy, whether provisional or a two-stent technique [6]. Interventional cardiologists are well aware of the difficulties that bifurcation disease can present during PCI. Not only can CCTA set the indication for PCI by detecting the presence of significant bifurcation disease, it can also aid in fully understanding the anatomy, plaque composition, and landing zones as well as in the assessment vessel dimensions [7,8].

Significant side branches at the site of coronary stenosis can influence the choice of stent length or type [9]. CCTA enables interventionalists to select the optimal stent size, contributing to better procedural outcomes. Two critical metrics derived from CCTA are the minimal lumen diameter and the reference vessel diameter. In clinical practice, the minimal lumen diameter is crucial for assessing lesion severity, while the reference vessel diameter, distal to the lesion, is used for selecting the stent diameter during percutaneous revascularization planning. For PCI planning, the CT-derived reference vessel diameter, measured at healthy coronary segments, can play a pivotal role in guiding stent size selection, contributing to more precise and effective intervention strategies.

Utilizing CCTA to assess coronary distributions relative to myocardial mass can help evaluate the potential benefits of revascularization and the risks associated with branch vessel occlusion [10,11]. For the indication of revascularization to side branches, the estimation of myocardial mass distal to the side branches and the measurements of side branch length by CCTA were proposed as useful in the decision making [12,13]. These findings help to decide whether we should protect the side branch or not and also which side branch should be protected among several side branches [14].

## 3. Coronary Plaque Assessment

**Figure 1:**
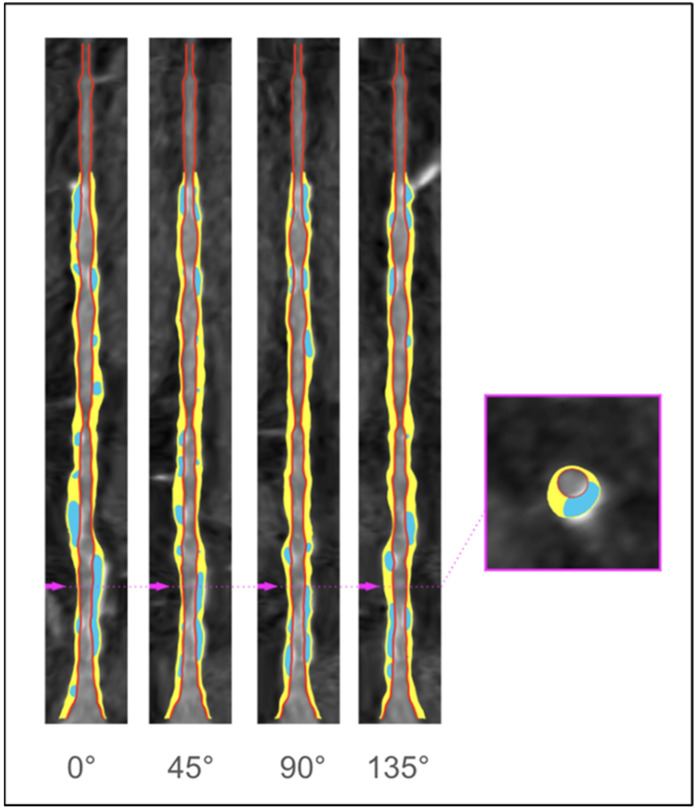
Artificial intelligence–enabled quantitative coronary plaque analysis (AI-QCPA) on coronary computed tomography angiography (CCTA). Blue-colored areas are calcified plaque, and yellow-colored areas are noncalcified plaque in the left anterior descending (LAD) coronary artery. The pink arrows of the longitudinal view (right) correspond to the specific corresponding cross-sectional view. CT images were analyzed using commercially available post-processing software iNtuition v4.4.13 (TeraRecon, Foster City, CA, USA).

**Figure 2:**
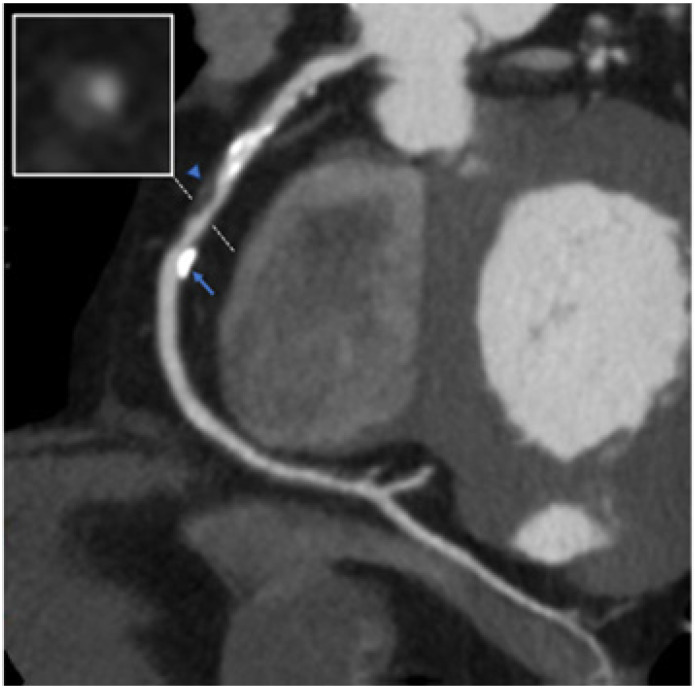
Coronary computed tomography angiography (CCTA) demonstrating 70–90% stenosis in the right coronary artery (RCA). A solid arrow indicates calcified plaque, while a blue triangular arrow highlights non-calcified plaque. A short-axis image (white dashed line) obtained using double oblique reconstruction shows plaque with high-risk features (napkin-ring sign).

CCTA provides complementary information to ICA regarding atherosclerotic plaque composition and volume, which could impact the invasive procedure as it is an imaging tool that allows a detailed evaluation of the extension, volume, and composition of atherosclerotic plaques, compared to conventional ICA, which only assesses coronary lumen narrowing (Figure 1). A primary goal for improving procedural success and patient outcomes is achieving complete plaque coverage. Incomplete plaque coverage of a diseased segment is associated with an increased risk of target-vessel revascularization and a threefold increase in myocardial infarction during the first year [15,16] (Figure 2). CCTA-based assessment of plaque extension enables the application of the normal-to-normal concept, as described with intravascular imaging. By positioning the stents on healthy coronary segments, the atherosclerotic plaque is covered, similar to the approach used with IVUS or OCT, which may help reduce the risk of a geographic miss [17,18].

**Figure 3:**
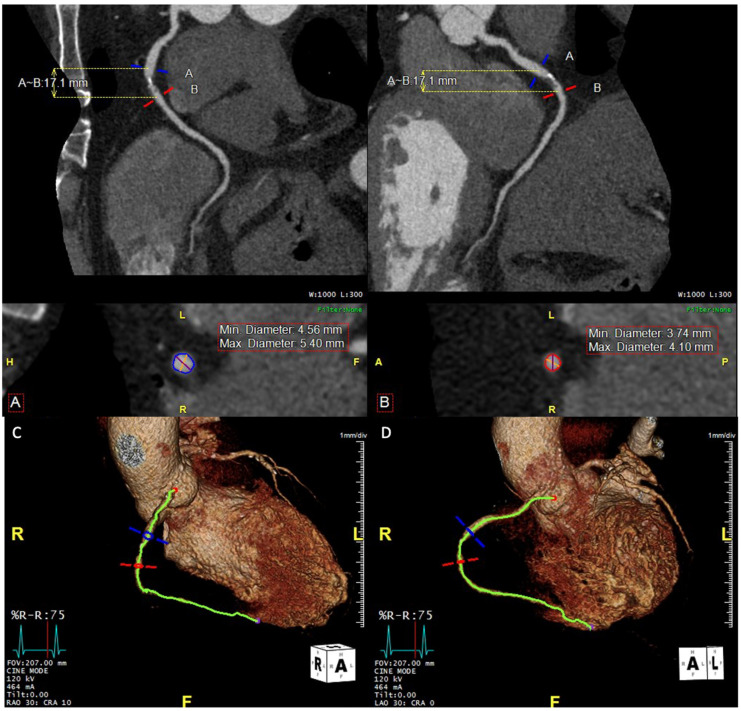
Coronary computed tomography angiography (CCTA) reveals severe stenosis in the mid-segment of the right coronary artery (RCA). (**A**) Red lines indicate the minimum diameter, and blue lines represent the maximum diameter of the proximal reference vessel. (**B**) Red lines show the minimum diameter, and blue lines indicate the maximum diameter of the distal reference vessel. A–B represents the lesion length. Volume-rendered CCTA images with fluoroscopic angles showing RCA stenosis: (**C**) RAO 30°, CRA 10°; (**D**) LAO 30°, CRA 0°. RAO: right anterior oblique; CRA: cranial; LAO: left anterior oblique.

The composition of plaque and the extent of coronary calcification can influence the planning process, particularly in determining whether plaque modification is necessary before stent placement [19]. The pre-procedural CCT has an advantage in demonstrating the overall picture of calcification clearly [20] (Figure 3), while IVUS, which is often used for the intra-procedural assessment in complex PCI, is not able to reveal the thickness of calcium. With a growing range of vessel preparation options, such as atherectomy, high-pressure balloons, intravascular lithotripsy, and drug-coated balloons, CCTA planning can enhance efficiency and help determine the optimal strategy for PCI. Additionally, the detailed morphological and plaque information provided by CCTA can guide decisions on the appropriate guidewire bending or the need for specialized techniques, such as the reverse wire technique, to navigate the guidewire into the side branch, as observed with OCT.

Coronary plaques can be classified as non-calcific, mixed, or calcific, based on the amount of calcium present in each lesion [21]. Calcified plaques are lesions with high CT attenuation (>320 HU), and highly calcified lesions are not suitable for the evaluation by CCT. Severely calcified coronary lesions may hinder stent delivery and, when not adequately treated, lead to stent under-expansion. Stent under-expansion is the most common cause of stent failure in the short- and mid-term follow-up [22]. Hence, visualization of high calcium burden at CCTA in the planning phase of coronary intervention may prompt use of calcium modification techniques (rotational atherectomy, orbital atherectomy, excimer laser, or intravascular lithoplasty) to facilitate stent expansion [23,24]. Also, the visualization of a concentric ring of calcium may prompt the interventionalist to consider lesion preparation to fracture the calcium plaque to facilitate stent expansion. By contrast, PCI with a high-pressure noncompliant balloon would be sufficient to achieve optimal stent expansion in the absence of concentric calcifications and short calcific plaque [25].

### Identifying High-Risk Plaque Features of CAD

CCTA also offers prognostic insights by detecting atherosclerosis, which can be used to stratify cardiovascular risk and guide medical therapy [26,27,28]. Coronary plaque composition plays a key role in predicting plaque stability; therefore, CCTA’s ability to accurately characterize coronary plaque features serves as a valuable tool for identifying patients at increased risk for peri- and postprocedural coronary events. Through both qualitative and quantitative analysis, CCTA identifies high-risk plaque features using parameters such as remodeling index and Hounsfield units (HU) [29]. CCTA can detect multiple high-risk plaque features, including positive remodeling (remodeling index of ≥1.1), low attenuation plaque (LAP, <30 Hounsfield Units), spotty calcification (focal calcification within the coronary artery wall <3 mm in maximum diameter), and napkin ring sign (low-attenuation plaque core with a rim of higher attenuation) [30,31,32,33]. Plaques with low HU have been identified as independent predictors of acute coronary syndromes, periprocedural myocardial infarction [34], and no-reflow phenomenon [35].

Nakazawa et al. reported that among 51 patients who underwent CCTA before PCI, the transient no-reflow phenomenon (absence of or slow coronary flow in the absence of significant coronary stenosis), which is associated with worse prognosis, was more common in those with napkin ring sign and LAP at CCTA [36]. Similarly, Watabe et al. demonstrated that the prevalence of high-risk plaque features identified at CCTA (LAP, positive remodeling, and spotty calcification) was higher among patients with post-PCI cTnT elevation >3 times the upper limit of normal [30]. High-risk plaque features have also been linked to myocardial ischemia as determined invasively. A sub-analysis of the NXT trial involving 254 patients showed that elevated low-attenuation plaque volume, quantified by CCTA, was independently associated with myocardial ischemia (fractional flow reserve (FFR) < 0.8), irrespective of lumen stenosis severity [35]. Further insights from the PARADIGM trial demonstrated that assessing coronary plaque progression using CCTA can identify plaques and patients at greater risk of disease progression. Among various metrics, percent atheroma volume (PAV), calculated as [(plaque volume/vessel volume) × 100], emerged as the most reliable predictor of progression from non-obstructive plaque to stenosis exceeding 50%. Changes in plaque volume, particularly of specific subtypes, can be used to monitor plaque progression or regression under medical therapy [37].

Non-calcified plaque and LAP have also been identified as independent predictors of low FFR [35]. Thus, even in cases of mild to moderate stenosis, the presence of high-risk plaques should prompt operators to consider invasive evaluations, such as FFR, to assess coronary lesions. Although further research is required to explore the clinical benefits of revascularizing high-risk plaques in the absence of significant stenosis, as well as the role of CCTA in identifying and guiding such decisions, current data support that advanced CCTA analysis of coronary atherosclerosis provides interventional cardiologists with critical information for guiding PCI.

## 4. Functional Evaluation of Coronary Stenoses

It is widely acknowledged that the relationship between the anatomical severity of coronary stenosis and its physiological impact is often inconsistent [38,39]. Current guidelines emphasize the importance of conducting invasive physiological assessments during ICA in symptomatic patients with high-risk clinical profiles and recommend considering revascularization to alleviate angina in patients with refractory symptoms despite optimal medical therapy [2].

**Figure 4:**
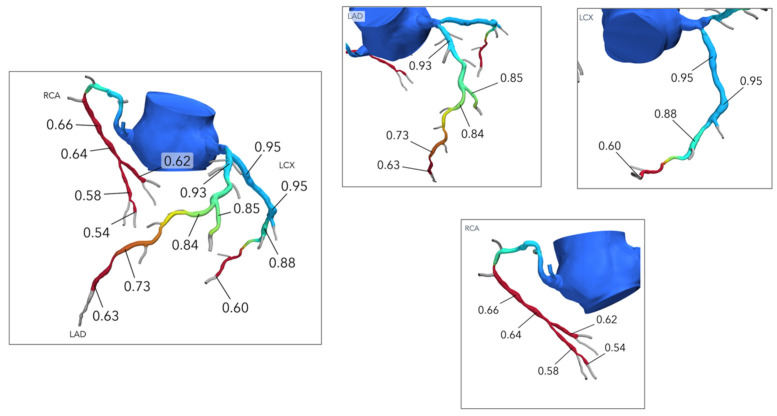
CT-derived fractional flow reserve (FFR-CT) model in the setting of a moderate proximal right coronary artery (RCA) lesion with an FFR-CT value of 0.64 at the distal RCA. FFR-CT analysis shows an abnormal translesional physiology in the distal left ascending aorta (LAD) due to a coronary lesion, with an FFR-CT value of 0.63 at the distal LAD.

New methods utilizing computational fluid dynamics and image-based modeling have been developed to calculate vessel-specific FFR-CT values from data routinely obtained through CCTA (Figure 4) [40]. Clinical trials have demonstrated improved diagnostic performance of FFR-CT in guiding ICA referral compared with a visual assessment for the detection of hemodynamically significant lesions [41]. FFR-CT provides the unique opportunity of achieving data on anatomy that includes lumen and plaque characteristics as well as lesion-specific physiology, combined information that is not provided by other non-invasive imaging modalities.

Functional evaluation with FFR-CT characterizes the pathophysiological pattern (focal or diffuse) of CAD noninvasively, visualized either on the color-coded geometry or by a virtual FFR-CT pull back curve. For example, patients with focal coronary lesions can exhibit a substantial pressure drop across a stenosis, but diffuse CAD may result in a slight, but constant, drop in pressure across the coronary vessel with decreased values only in the distal segments [42]. Τhe Assessing Diagnostic Value of Noninvasive FFR-CT, or ADVANCE registry, highlighted the significance of the delta FFR-CT or translesional gradient in the discrimination of patients who underwent early revascularization compared with a standard diagnostic strategy of CCTA with FFR-CT [43]. Thus, FFR-CT offers the opportunity to integrate information such as the lesion location, the depth of the physiologic disturbance, and the translesional gradient into the PCI planning process in contrast to angiographic anatomy evaluation, which cannot discriminate the physiologic phenotype [44].

Moreover, an FFR-CT planner tool (HeartFlow Planner; HeartFlow Inc., Redwood City, CA, USA) has recently been developed that predicts the results of PCI in terms of post-PCI FFR by simulating the luminal changes produced by PCI and recalculating coronary pressures. PCI that restores vessel physiology in focal functional CAD can provide clinical benefits by alleviating angina. Greater improvements in FFR following PCI are associated with more significant symptomatic relief and a reduced incidence of vessel-oriented clinical events [45,46,47]. In contrast, it is speculated that diffuse functional CAD is the most frequent cause of low improvement in FFR after revascularization, and suboptimal post-PCI results are plausibly responsible for persistent angina after PCI. Therefore, cases of anatomically diffuse CAD are often managed conservatively with optimal medical treatment or coronary artery bypass graft (CABG) [48]. The prospective Precise Percutaneous Coronary Intervention Plan (P3) study recently showed that the FFR-CT planner tool has high precision and accuracy in predicting FFR after PCI, with good agreement with invasive post-PCI FFR measurement, independent of image quality and CAD complexity [49].

## 5. Cardiac Computed Tomography-Derived Coronary Artery Volume to Myocardial Mass

According to current data, the prevalence of obstructive CAD in patients undergoing ICA does not exceed 33% [2]. An important subset of these patients includes patients suffering from ischemia with non-obstructive coronary artery disease (INOCA). INOCA is defined as (a) clinical symptoms associated with myocardial ischemia, (b) the absence of obstructive CAD (<50% diameter stenosis or FFR > 0.80), and (c) objective evidence of myocardial ischemia [50,51]. The major mechanism of INOCA is vasospasm of the epicardial coronary artery and coronary microvascular dysfunction, including microvascular spasm, an increase in microvascular resistance, the slow flow phenomenon, and microvascular vasodilatory dysfunction [50].

The evidence supporting physiology-guided assessment of coronary stenoses using invasive FFR has established that the diameter stenosis from ICA is not enough in itself to identify significant CAD [52]. Additionally, the effect of diffuse atherosclerotic plaque on the vasodilatory capacity of epicardial vessels may not be fully captured by ICA. Moreover, patients with isolated microvascular dysfunction, without evidence of CAD, can experience angina and myocardial perfusion abnormalities [53]. These limitations have contributed to the growing recognition of the need for a more integrated approach to assessing physiological supply versus demand.

The coronary artery lumen volume-to-myocardial mass ratio (V/M) bridges the gap between the epicardial coronary arteries and the underlying myocardium, offering valuable insights into the ability of the coronary arteries to meet myocardial demand. CCTA is uniquely positioned to non-invasively measure the V/M ratio. A standard CCTA acquisition offers the ability to extract both a detailed patient-specific three-dimensional model of the coronary geometry and an accurate volumetric assessment of left ventricular mass [11].

First, the coronary artery luminal boundaries are segmented from the CCTA images using deep learning-based centerline and lumen boundary analysis methods. These techniques trace the paths of the epicardial coronary arteries down to approximately 1 mm in diameter from the extracted imaging data [54], and the process typically takes only a few minutes. Semi-automated luminal segmentation using standard CT image processing software is also possible [55]. Once the vessel centerline tree is determined, the coronary luminal boundary is segmented, and the coronary luminal volume (V) inside the surface is calculated. Next, the left ventricular myocardial mass (M) is computed. The LV myocardial volume is segmented from cardiac CT images (area × slice thickness), and this volume is then converted to LV mass by multiplying with an assumed tissue density of 1.05 g/cc. Finally, by dividing the coronary artery luminal volume (V) by the LV myocardial mass (M), the V/M ratio is calculated. The refinement of this quantification method is based exclusively on the CT-derived epicardial coronary lumen volume from vessels greater than 1 mm in diameter [54].

The linear relationship between vessel volume and myocardial mass can be disrupted in coronary artery diseases such as endothelial dysfunction, diffuse intimal thickening, and arteriosclerosis, as well as in myocardial conditions like aortic stenosis or hypertrophic obstructive cardiomyopathy. This relationship may also be influenced by factors like blood pressure, heart rate, and hypoxia. Since the V/M ratio serves as an integrated measure of resting myocardial blood supply and demand in vivo, it has the potential to provide valuable insights into diseases where this balance is disturbed, thus offering new perspectives for clinical diagnoses and management [11].

The coronary volume-to-mass ratio is a promising investigational tool that provides insights into vascular health and the mechanisms of abnormal physiology. When combined with FFR-CT, the V/M ratio could offer additional non-invasive information to enhance our understanding of CAD mechanisms. With this concept established, future studies should focus on determining whether V/M can be modified through medical management and cardiac rehabilitation and whether improvements in the V/M ratio are linked to better clinical outcomes.

## 6. Cardiac Computed Tomography-Derived Myocardial Viability

The viability of the myocardium distal to an occluded lesion is a critical factor in determining the need for revascularization, especially for chronic total occlusion (CTO) lesions. While cardiac magnetic resonance imaging (MRI) and the combination of positron emission tomography (PET) with CCT are considered the gold standards for assessing myocardial viability [56,57,58,59], CCTA can also identify significant myocardial damage, such as left ventricular (LV) myocardial thinning. Additionally, several studies have shown that resting CCT perfusion has a high sensitivity and specificity for detecting prior myocardial infarction, ranging from 77% to 91% and 79% to 97%, respectively, compared with the reference standard of MRI [60].

CT-MPI contributes to the diagnostic accuracy of functional assessments in CAD, with performance metrics similar to CT-based FFR. Both functional applications of CT provide complementary diagnostic value, with CT-MPI being particularly useful in patients with extensive coronary calcification, chronic occlusions, or a history of previous revascularization. The sensitivity and specificity of CT-MPI for detecting functionally significant CAD range from 82% to 92% and 73% to 86%, respectively, when compared to invasive FFR [61].

In clinical practice, CT-MPI can be used following a positive CCTA to assess the functional severity of coronary lesions, offering the advantage of direct spatial correlation between stenotic branches and the myocardium they supply. The myocardial mass distal to the culprit lesion can be easily calculated with current CT software programs, making pre-procedural assessment by CCTA valuable for evaluating myocardial viability and ischemic burden. This is particularly important when deciding whether to proceed with PCI in CTO lesions, although the exact cutoff value for the amount of viable myocardial mass remains unclear [62,63].

Stress CT-MPI has emerged as a promising technique that combines both anatomical and functional evaluation in a single procedure. Several studies have demonstrated that CT-MPI enhances the diagnostic accuracy of CCTA in detecting obstructive CAD. However, only a few small studies have explored the potential value of cardiac CT incorporating CT-MPI in patients with coronary stents, with results suggesting that a combined CCTA and CT-MPI protocol improves diagnostic accuracy compared to CCTA alone [64].

## 7. Cardiac Computed Tomography in Special Clinical Scenarios

### 7.1. Coronary Anomalies

When coronary anomalies are discovered during invasive heart catheterization, performing a CCTA afterward can help fully elucidate the true course and potential risks of the anomaly [65,66]. This post-procedure CCTA provides additional anatomic detail, helping clinicians better understand the anomaly’s features and implications for treatment. Additionally, coronary anomalies identified on CCTA can facilitate a quicker and more precise engagement of the coronaries in the cath lab. The detailed pre-procedural imaging enables better navigation of anomalous coronary anatomy, in contrast to cases where the coronary origin is unknown. This advanced knowledge improves procedural efficiency and helps reduce the risk of complications [65,66].

### 7.2. Coronary Fistula

A coronary fistula refers to an abnormal communication between the coronary arteries and parts of the pulmonary or systemic circulation [67]. While conventional coronary angiography remains critical in evaluating coronary fistulas, CCTA has become the preferred noninvasive imaging technique due to its high spatial resolution. With multiplanar reconstruction and 3D volume-rendered images, CCTA allows detailed assessment of the fistula’s origin, course, and drainage site, as well as the surrounding cardiac and vascular structures. This makes CCTA an invaluable tool for preprocedural planning and clinical decision making.

### 7.3. Coronary Aneurysm

A coronary artery aneurysm is defined as a dilatation of the coronary artery more than 1.5 times the normal size of the adjacent vessel. While ICA provides essential information about the location, shape, and atherosclerosis of the aneurysm, it cannot reliably assess the aneurysm’s size in the presence of thrombi, as it is limited to luminal assessment [43]. CCTA, however, provides high-quality multiplanar and 3D imaging, offering accurate details regarding the shape, diameter, plaque composition, and presence of concomitant stenosis, as well as the aneurysm’s relationship to nearby cardiac structures. CCTA is instrumental in determining the anatomic characteristics of coronary aneurysms and provides a roadmap for interventional cardiologists during PCI.

### 7.4. Post-CABG

Following coronary artery bypass graft (CABG) surgery, particularly in patients experiencing chest pain, determining graft patency is crucial. CCTA is an excellent tool for assessing graft patency, as it can accurately visualize larger caliber grafts (especially venous grafts) with less susceptibility to motion artifacts compared to coronary arteries [68]. CCTA is useful in evaluating grafts in patients with recurrent chest pain or abnormal findings on functional cardiac testing that suggest ischemia in a specific coronary bypass graft. It also provides critical insights into disease progression, graft anatomy, and the location and topography of grafts before proceeding with ICA. Volume-rendered images of CCTA are particularly beneficial in complex CABG cases, aiding in identifying graft origin, course, and termination. When ICA is necessary, pre-procedural CCTA can help save radiation, contrast, and time by giving operators clear insight into graft locations and patency [69]. The benefits of CCTA in post-CABG patients, particularly in terms of improving procedural efficiency and reducing radiation and contrast dose, have been demonstrated in a randomized controlled trial (RCT). The CORE320 study specifically evaluated the use of CCTA in the follow-up of patients who had undergone CABG. The trial showed that CCTA was effective in detecting graft failure and assessing coronary anatomy, offering a non-invasive alternative to traditional ICA. Additionally, it highlighted how CCTA could significantly reduce both radiation exposure and contrast agent use, thereby improving patient safety and comfort during follow-up evaluations. This advanced imaging approach ultimately enhanced procedural efficiency by providing more precise and comprehensive anatomic information without the need for repeated invasive procedures [70].

### 7.5. Chronic Total Occlusion (CTO)

PCI for CTO remains a challenging procedure, with success rates often under 90% in daily clinical settings [71,72,73,74,75,76]. Recent high-quality evidence from RCTs, such as the CTO-CT trial, highlights the significant benefits of CCTA in predicting procedural success for CTO PCI. This trial specifically demonstrated that CCTA guidance improves procedural outcomes by providing comprehensive anatomic insights, which enhance both the safety and efficacy of hybrid CTO PCI strategies. These detailed imaging capabilities allow for more precise planning and execution, ultimately leading to better success rates and reduced complications during the intervention [77]. CCTA has become a crucial step in evaluating the risk-to-benefit ratio for CTO PCI procedures. Recent data suggest that CCTA can facilitate PCI in CTO cases by providing comprehensive anatomic information about the entire coronary vasculature. CCTA can detail the structure of atherosclerotic plaques and the characteristics of CTO segments, including entry and exit morphologies, occluded route, vessel diameter, and presence of calcification [78]. Understanding the course of the vessel can guide both antegrade and retrograde approaches and inform crossing strategies based on the characteristics of the proximal and distal atherosclerotic caps. Most centers now use CCTA for particularly complex CTO cases, and integrating CCTA as a routine preprocedural tool for CTO PCI planning—and incorporating CCTA images into the catheterization laboratory as reference or fusion data—can further improve procedural strategies and outcomes [43] Several scoring systems derived from CCTA have been developed as accurate, non-invasive tools for predicting the procedural success of hybrid CTO PCI [79].

## 8. Diagnostic Advances and Limitations of CCTA in Obstructive CAD

Based on recent studies, CCTA has shown significant promise in diagnosing obstructive CAD. A meta-analysis conducted by Schlattmann et al. [80] demonstrated the effectiveness of CCTA in diagnosing obstructive CAD, particularly when combined with functional testing. This approach has been found to provide accurate results, with high diagnostic performance in both symptomatic patients and those with stable chest pain, as evidenced by the individual patient data analysis in the Collaborative Meta-Analysis of Cardiac CT (COME-CCT) [80]. Similarly, a prior meta-analysis by Haase et al. [81] emphasized the role of CTA in the diagnosis of obstructive CAD, highlighting its effectiveness in varying clinical settings, including low- and high-probability cases of CAD [81]. However, despite these advantages, the use of CCTA in the catheterization laboratory presents certain challenges. One of the primary barriers is the potential increase in overall diagnostic costs. As CTA often requires advanced imaging technology, its integration with ICA can lead to significantly higher procedural expenses. Additionally, the combined use of both CCTA and ICA increases the contrast load administered to patients, which may raise concerns regarding contrast-induced nephropathy, particularly in individuals with compromised renal function. These factors underscore the need for careful patient selection and appropriate management strategies to mitigate the risks associated with contrast use.

## 9. Future Directions

### 9.1. Artificial Intelligence, Machine Learning, and Radiomics

Incorporating artificial intelligence (AI), machine learning (ML), and radiomics into daily clinical practice holds great potential to enhance diagnostic accuracy and reduce the time needed for CCTA analysis. Given the operator-dependent and time-consuming nature of assessing plaque characteristics, it is clear that integrating AI and ML algorithms for automatic identification and reporting of such features could revolutionize clinical practice. Recent studies underscore AI’s growing role in coronary imaging. For example, Griffin et al. [82] showed that AI-based assessment of stenosis severity on CCTA closely matched quantitative coronary angiography and FFR, highlighting AI’s accuracy in evaluating plaque and stenosis. Additionally, AI algorithms can predict ischemia by analyzing quantitative CCTA parameters, helping identify functionally significant lesions that might not be visible on standard imaging. This is further supported by Choi et al. [83], whose CLARIFY study demonstrated AI’s ability to assess atherosclerosis, stenosis, and vascular morphology, facilitating more efficient coronary disease management. In the ISCHEMIA trial [84], AI-guided quantitative coronary computed tomography angiography (AI-QCT) was shown to predict outcomes by accurately quantifying atherosclerosis and assessing cardiovascular risk, aiding in risk stratification and personalized treatment strategies. Furthermore, a study by Ihdayhid et al. [85] validated AI-based plaque quantification from CCTA, showing it closely matched the diagnostic performance of IVUS in plaque characterization and stenosis assessment. These technological advancements would enable quantitative measurements of coronary plaques, significantly improving diagnostic workflows and providing insights into clinical decision making [86,87,88].

### 9.2. Advancements in CT Technology for PCI Guidance

In addition to AI and radiomics, improvements in CT technology can further enhance PCI guidance. Photon-counting CT (PCCT), the latest advancement in CT scanning, offers several advantages over traditional CT scanners. PCCT provides high-resolution imaging, improved contrast resolution, and simultaneous multi-energy imaging, offering superior capabilities in cardiovascular imaging compared to traditional dual-energy CT. Photon-counting detectors work by directly converting X-ray photons into electrical signals, improving CT dose efficiency and achieving ultra-high resolution (0.2 mm). This advancement significantly benefits the assessment of calcified coronary plaques or coronary stents, reducing noise and artifacts and allowing for more precise imaging. PCCT has been shown to improve spatial resolution, enabling better detection of stented areas, small coronary branches, and calcification compared to conventional CT technology [89,90,91]. A recent study [92] highlighted the enhanced ability of PCCT to assess stenosis severity in calcified lesions. Traditional CT scans often face challenges in accurately estimating stenosis in the presence of significant calcification, as dense calcium can obscure the vessel lumen. The high-resolution and multi-energy capabilities of PCCT, however, have been shown to reduce these artifacts, enabling a more accurate quantification of stenosis severity, particularly in heavily calcified coronary segments. Another trial [93] investigated the role of PCCT in stent evaluation, particularly in detecting restenosis and neo-intimal hyperplasia. The study demonstrated that PCCT provides superior contrast resolution compared to conventional dual-energy CT for detecting small-scale changes in stented coronary segments, such as early restenosis, stent under-expansion, and stent thrombosis. Early studies with PCCT in CCTA have demonstrated improvements in calcium volume and Agatston score [94] highlighting its enhanced ability to identify both calcified and non-calcified plaques, including lipid-rich and fibrotic plaques [94,95]. However, the broader clinical impact of this technology, particularly its ability to identify vulnerable plaques, remains unclear and warrants further investigation.

### 9.3. The P4 Study and Beyond

The ongoing Precise Procedure and PCI Planning and Guidance (P4) study aims to compare CCTA-guided PCI with IVUS-guided PCI. The results of this study are expected to provide a foundation for integrating CCTA into routine coronary interventions. Additionally, the development of software that allows online co-registration and simulates cardiac cycle movement may further enhance the synergy between CCTA and IVUS, expanding their combined utility in procedural planning and guidance [11].

## 10. Conclusions

CCTA is rapidly becoming the gateway to the catheterization laboratory, making it essential for interventional cardiologists to incorporate CCTA findings into the planning of interventions. By integrating CCTA into the procedural planning for patients referred for invasive evaluation, it has the potential to streamline the course of ICA, improve diagnostic accuracy, optimize therapeutic interventions, and ultimately enhance clinical outcomes.

## Data Availability

No new data were created, as this is a review article.
